# Fat and lean mass changes during incretin-based therapy with structured exercise and nutritional support: A retrospective real-world pilot study

**DOI:** 10.1016/j.obpill.2026.100335

**Published:** 2026-09-19

**Authors:** Meral Kucuk Yetgin, Nana Gletsu-Miller, Robert R. Chapman, Eitan Gelber, John Snyder, Donny Gregg, Dustin Nabhan

**Affiliations:** a Department of Applied Health Science, School of Public Health - Bloomington, Indiana University, Bloomington, IN, USA; b Department of Kinesiology, School of Public Health - Bloomington, Indiana University, Bloomington, IN, USA; c Apeiron Life, Menlo Park, CA, USA; d Cleveland Browns, Berea, OH, USA

**Keywords:** Body composition, Exercise, Glucagon-like peptide-1 receptor agonist, Lean mass, Nutritional support, Obesity

## Abstract

**Background:**

Incretin-based therapies are highly effective for weight reduction, but changes in lean mass during treatment remain a clinical concern. This study evaluated changes in fat mass and lean mass in individuals receiving structured exercise and nutritional support during incretin-based therapies.

**Methods:**

In this single-center retrospective observational chart-review study, 11 adults who underwent 9–12 months of incretin-based therapy and were offered a program providing supervised exercise and individualized nutritional support were analyzed. No control group was included. Treatments included glucagon-like peptide-1 (GLP-1) receptor agonists and glucose-dependent insulinotropic polypeptide (GIP)/GLP-1 receptor co-agonists. Body composition was assessed using dual-energy X-ray absorptiometry (DXA). Data were assessed for parametric assumptions, and pre- and post-intervention comparisons were performed using paired-samples t tests.

**Results:**

Significant reductions were observed in body weight (mean change: −4.99 kg [95% CI: −7.65 to −2.34]; p = 0.002) and fat mass (−4.37 kg [95% CI: 6.36 to −2.39]; p < 0.001), corresponding to a mean total weight reduction of 4.71% and a mean fat mass reduction of 11.06%**,** respectively. Lean mass did not change significantly (−0.62 kg [95% CI: 2.20 – 0.95]; p = 0.397). At the aggregate cohort-level, weight reduction composition analysis indicated that 87.58% of total weight reduction was attributable to fat mass, and 12.42% to lean mass.

**Conclusion:**

Incretin-based therapy delivered within a structured exercise and nutritional support program was associated with fat-dominant weight reduction without a statistically significant change in lean mass. These findings are hypothesis-generating and warrant confirmation in adequately powered prospective controlled studies.

## Graphical abstract

**Figure:**
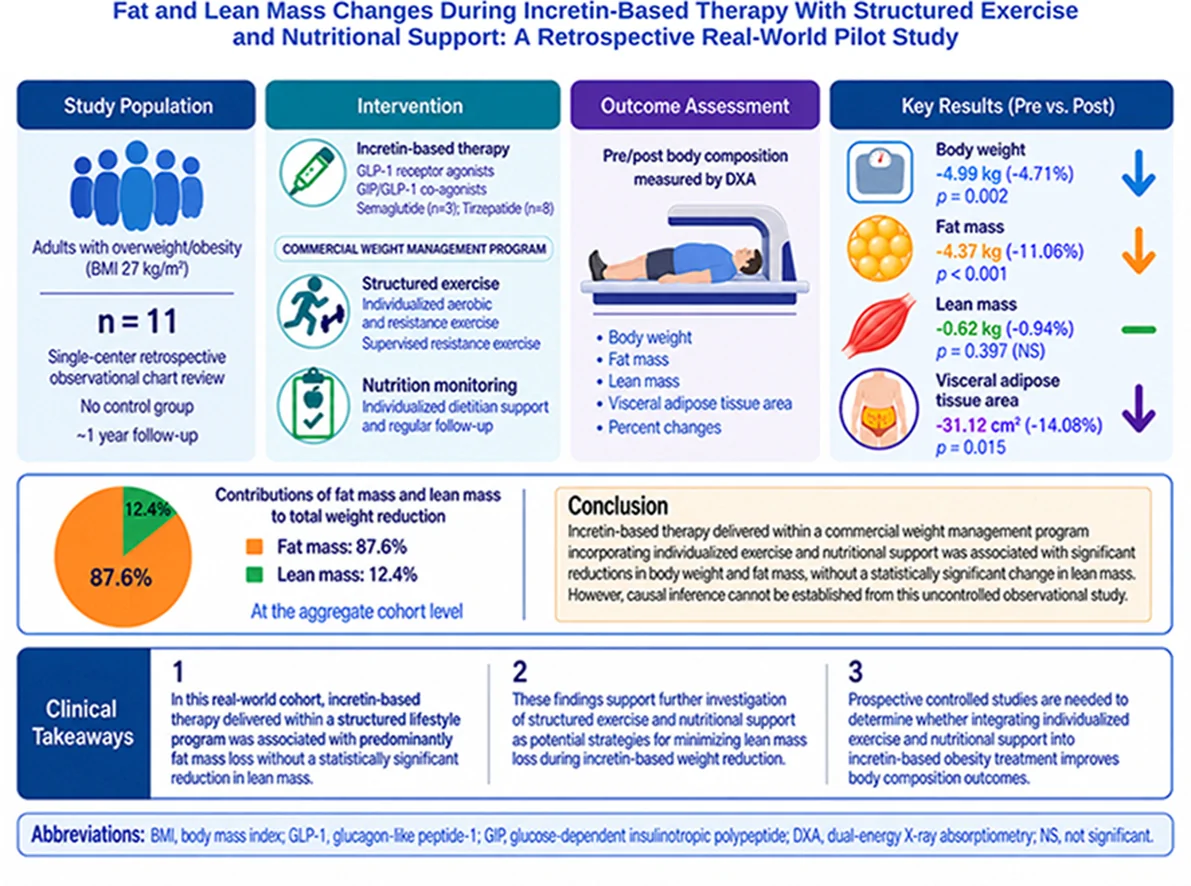

## 1 Introduction

Obesity is a chronic, multifactorial metabolic disease requiring long-term management and is associated with increased health risks and numerous comorbidities [1]. The improved efficacy and safety of obesity medications have established pharmacotherapy as an important second-line treatment option for obesity management [2]. Incretin-based therapies, including glucagon-like peptide-1 (GLP-1) receptor agonists and glucose-dependent insulinotropic polypeptide (GIP)/GLP-1 receptor co-agonists, are among the most effective pharmacological treatments currently available for obesity. Among these agents, semaglutide and tirzepatide produce substantial and sustained weight reduction. The head-to-head SURMOUNT-5 trial demonstrated greater weight reduction with tirzepatide than with semaglutide, with mean weight reductions of 20.2% and 13.7% [3]. However, body composition analyses of semaglutide and tirzepatide have shown reductions in both fat and lean body mass [4,5]. Importantly, lean mass may constitute a substantial proportion of total weight reduction during incretin-based therapy. In the STEP 1 body composition analysis, semaglutide reduced body weight by 15.0%, fat mass by 19.3%, and lean body mass by 9.7% [6]. Similarly, the SURMOUNT-1 DXA substudy showed that tirzepatide reduced body weight by 21.3%, fat mass by 33.9%, and lean mass by 10.9%, with 26% of total weight reduction occurring as lean mass loss [7]. A meta-analysis reported lean mass contributions of 35.2% for semaglutide, 25.4% for tirzepatide, and 26.8% for liraglutide [8].

In contrast, studies incorporating structured exercise have demonstrated improved weight reduction maintenance, although their effects on preserving lean mass during weight reduction have been less extensively investigated [9,10]. Recent clinical guidance and reviews emphasize the integration of pharmacotherapy with structured exercise and appropriate nutritional strategies [[11], [12], [13]]. Specific recommendations include adequate protein intake of approximately 1.2–1.6 g/kg/day [14], resistance training 2–3 times per week, and at least 150 min of moderate-intensity aerobic exercise per week [[14], [15], [16]]. In real-world clinical practice, however, most patients receive incretin-based therapy through office-based care with limited access to individualized nutritional counseling and supervised exercise programs [5,17]. A recent network meta-analysis found greater reductions in body weight and fat mass with combined GLP-1 receptor agonist and structured exercise therapy than either approach alone, while insufficient data precluded analysis of lean mass [18]. Although prospective real-world studies such as SEMALEAN [19] have reported DXA-derived body composition changes during incretin-based therapy, to the best of our knowledge, no previous real-world study has evaluated DXA-derived body composition in patients initiating incretin-based therapy while simultaneously being offered individualized exercise prescription and dietitian-delivered nutritional support. Therefore, this pilot real-world study aimed to evaluate changes in fat and lean mass among individuals receiving incretin-based therapy within a commercial weight management program providing individualized exercise prescription and nutritional support, with a particular focus on lean mass changes.

## 2 Methods

This was a single-center retrospective observational chart-review study of records from individuals participating in a commercial weight management program between 2022 and 2025. A total of 25 individuals receiving incretin-based therapy in combination with a structured exercise program and individualized nutritional support were screened for study eligibility. Seven participants were excluded due to the absence of a post-intervention DXA assessment, one participant was excluded because the follow-up DXA scan was performed more than one year after the intervention, and six participants were excluded because their BMI did not meet the study eligibility criteria. Consequently, the final analytical cohort consisted of 11 participants who met the study eligibility criteria and were included in the analysis. Inclusion criteria were: (1) BMI ≥27 kg/m^2^ [20] (2) concurrent initiation of incretin-based therapy and the structured exercise and nutrition program, (3) approximately 1 year of follow-up within the program, (4) availability of pre- and post-intervention dual-energy X-ray absorptiometry (DXA) assessments, and (5) documented participation in the prescribed exercise program based on trainer records and follow-up documentation maintained within the program, (6) documented participation in individualized nutritional support, assessed through interviews, questionnaires, and regular follow-up consultations with the dietitian.

Exclusion criteria included a history of bariatric surgery. Data were extracted from the electronic records, including demographics (age, sex), medical history, medication use, and incretin-based therapy characteristics. Anthropometric measures included body weight (BW), height, waist circumference (WC), and body mass index (BMI). Body composition was assessed using a dual-energy X-ray absorptiometry (DXA) system (Horizon W, Hologic Inc., Marlborough, MA, USA). Whole-body lean mass (LM), fat mass (FM), total body fat percentage (TBF), visceral adipose tissue (VAT) area, fat mass index (FMI), lean mass index (LMI), and bone mineral content (BMC) were obtained from the DXA analysis. LM corresponded to the DXA-derived Lean + BMC measure and therefore included BMC. Appendicular lean mass (ALM) was calculated as the sum of lean mass from both arms and both legs, and appendicular lean mass index (ALMI) was calculated as ALM divided by height squared (kg/m^2^). Scans followed manufacturer procedures, including routine quality control and calibration. A facility-specific least significant change (LSC) assessment had not been established for the DXA system. In routine clinical practice at the facility, a threshold of 1.4% or 2 lb was used for longitudinal soft-tissue changes.

The cohort was treated with semaglutide (Novo Nordisk A/S, Bagsværd, Denmark; n = 3) or tirzepatide (Eli Lilly and Company, Indianapolis, IN, USA; n = 8). Participants underwent medical clearance to undergo an exercise program and a baseline fitness assessment and optimal heart rate zones using a graded treadmill exercise test according to the Balke protocol [21].

Participants were offered individualized exercise programs prescribed by certified specialists in accordance with guideline-based recommendations. The prescribed targets included 150–300 min/week of moderate-intensity or 75–150 min/week of vigorous-intensity aerobic exercise and ≥2 sessions/week of supervised resistance training at 60–70% of one-repetition maximum (1-RM) [15]. Actual participation in supervised resistance training was retrospectively determined from trainer records and is reported in the Results. Resistance training incorporated machine-based and free-weight exercises, mobility and stability training, with individualized progressive overload through adjustments in repetitions, sets, intensity, and exercise complexity based on performance and tolerance. Aerobic exercise was recommended and was generally performed outside direct staff supervision, with adherence assessed differently from supervised resistance training. Nutritional guidance was individualized for each participant, with protein intake recommended to be obtained primarily from whole-food sources rather than supplements, including lean fish, seafood, poultry, dairy products, and legumes [14]. Protein intake and dietary compliance were assessed by dietitians using 24-h dietary recalls during follow-up. Participants were monitored by trainers and dietitians through interviews, questionnaires, and regular communication/check-ins conducted either in person or virtually, with optional remote tracking when appropriate.

Statistical analyses were performed using JASP (JASP Team (2026). JASP (Version 0.98.0) [Computer software]) and RStudio software (Posit team (2025), Posit Software, PBC, Boston, MA, USA) [22]. Descriptive statistics for continuous variables presented with mean and standard deviation (SD) when data met parametric assumptions, otherwise reported with median and interquartile range (IQR; Q25–Q75). Categorical variables presented with frequency (percent). The distributions of independent group differences and paired pre-post differences were assessed using graphical methods, Shapiro-Wilk test, and/or Levene's test. Baseline characteristics between the study group and excluded participants compared with Fisher's Exact test, independent samples *t*-test, Mann-Whitney test, and Welch's test. Study group's pre- and post-intervention differences compared with paired samples *t*-test. The primary outcomes were BW, FM, and LM, while the remaining body composition measures were treated as exploratory secondary outcomes. As a sensitivity analysis for multiplicity, the Benjamini-Hochberg (BH) procedure was applied across all 12 pre-post comparisons to control the false discovery rate at 0.05. BH-adjusted p-values are reported in Table 2. The mean differences reported with 95% confidence interval (CI), and percentage change was calculated as [(post-intervention value − pre-intervention value)/pre-intervention value] × 100. The aggregate cohort-level contributions of fat mass and lean mass to total weight reduction were calculated proportionally from the [mean ΔFM/mean ΔBW] × 100, and [mean ΔLM/mean ΔBW] × 100, where ΔBW = ΔFM + ΔLM. Effect sizes reported as Cohen's d (small: 0.2–0.5, medium: 0.5–0.8, large: d ≥ 0.8) [23] and Rank biserial correlation (no relationship: 0, study group > excluded participants: r > 0, study group < excluded participants: r < 0) [24]. Correlation relationship between change in body weight and change in lean mass was investigated with Pearson's correlation. Statistical significance was set at p < 0.05 level.

## 3 Results

A total of 11 participants completed the intervention and were included in the final analysis, comprising 2 women (18.2%) and 9 men (81.8%). Recorded comorbidities included dyslipidemia, hypertension, hypothyroidism, type 2 diabetes, autoimmune disease, and obstructive sleep apnea (OSA). Concomitant therapies included lipid-lowering, antihypertensive, thyroid hormone replacement, and glucose-lowering medications; one participant used continuous positive airway pressure (CPAP) for OSA. The mean age of the participants was 53.46 years (SD:12.32) ranging from 34 to 74 years, and the median baseline BMI was 28.7 kg/m^2^ (IQR: 28.4–33.9). At the final body composition assessment, the documented weekly doses ranged from 1 to 2.4 mg/week for semaglutide and from 2.5 to 12.5 mg/week for tirzepatide. Participants completed a mean of 41.36 ± 21.50 supervised resistance-training sessions per participant during follow-up (range, 2–73), corresponding to 1.24 ± 0.50 sessions/week (range, 0.60–2.00). Eight participants showed partial dietary compliance (72.7%), while three participants were classified as non-compliant (27.3%). Protein intake per kilogram increased significantly by 53.33% after the intervention (pre: 0.75 ± 0.55 g/kg/day versus post: 1.15 ± 0.36 g/kg/day; mean difference [95% CI] = 0.40 [0.17–0.63] g/kg/day; p = 0.003; d = 1.17). The median follow-up duration was 277 days (IQR, 236–328 days).

The baseline characteristics between the study group and the excluded participants were not significantly different in terms of sex, age, TBF percentage, and BMC (p > 0.05) (Table 1). The study group had significantly higher BW, BMI, WC, FM, FMI, VAT, LM, LMI, ALM and ALMI compared to excluded participants (p < 0.05) (Table 1).

**Table 1:** Baseline characteristics of the study group and excluded participants.

	Study Group (n = 11)	Excluded participants (n = 14)	p	Effect size
	Statistic	Statistic		
**Sex, Male**	9 (81.82)	10 (71.43)	0.661^a^	NA
**Age (years)**	53.46 (12.32)	54.50 (11.53)	0.829^b^	0.088
**BW (kg)**	105.93 (24.41)	80.50 (18.20)	0.007^b^*	1.204
**BMI (kg/m^2^)**	28.70 (28.38–33.93)	25.03 (23.39–28.43)	0.004 ͨ*	0.682
**WC (cm)**	115.70 (18.55)	99.08 (14.19)	0.018^b^*	1.024
**TBF (%)**	36.64 (7.73)	31.75 (6.79)	0.106^b^	0.677
**FM (kg)**	36.02 (31.30–40.74)	24.22 (18.87–28.81)	0.011 ͨ*	0.597
**FMI (kg/m^2^)**	10.20 (9.50–12.70)	8.15 (6.85–9.40)	0.017 ͨ*	0.571
**VAT area (cm^2^)**	221.03 (111.12)	127.49 (45.94)	0.021^d^*	1.100
**LM (kg)**	66.42 (13.34)	54.73 (12.18)	0.032^b^*	0.921
**LMI (kg/m^2^)**	18.86 (2.49)	16.64 (2.24)	0.028^b^*	0.947
**ALM (kg)**	28.92 (6.33)	23.35 (5.93)	0.033^b^*	0.912
**ALMI (kg/m^2^)**	8.62 (1.24)	7.45 (1.26)	0.029^b^*	0.939
**BMC (kg)**	3.26 (0.71)	2.78 (0.64)	0.092^b^	0.709

**Abbreviations:** BW, body weight; BMI, body mass index; WC, waist circumference; TBF, total body fat; FM, fat mass; FMI, fat mass index; VAT area, visceral adipose tissue area; LM, lean mass (includes BMC); LMI, lean mass index; ALM, appendicular lean mass; ALMI, appendicular lean mass index; BMC, bone mineral content; NA, not applicable; SD, standard deviation; IQR, interquartile range. Descriptive statistics presented with mean (SD) except for sex presented with n (%), and the variables of BMI, FM and FMI are presented with Median (IQR). Baseline characteristics between the study group (n = 11) and excluded participants (n = 14) compared with ^a^Fisher's Exact test, ^b^Independent Samples *t*-test (Effect size: Cohen's d), ͨ Mann-Whitney test (Effect size: Rank biserial correlation), and ^d^Welch's test (Effect size: Cohen's d). *Statistically significant difference at p < 0.05 level (two-tailed).

**Table 2:** Changes in body composition parameters following the intervention (n = 11).

	Pre	Post	Mean difference (95% CI)	p	BH adjusted p	Cohen's d
**BW (kg)**	105.93 (24.41)	100.94 (23.62)	−4.99 (−7.65–−2.34)	0.002*	0.0056*	1.263
**BMI (kg/m^2^)**	31.70 (5.80)	30.18 (5.51)	−1.52 (−2.37–−0.68)	0.002*	0.0060*	1.207
**WC (cm)**	115.70 (18.55)	113.16 (16.64)	−2.54 (−5.11–0.03)	0.052	0.0890	0.665
**TBF (%)**	36.64 (7.73)	34.28 (7.88)	−2.36 (−3.56–−1.15)	0.001*	0.0056*	1.310
**FM (kg)**	39.51 (16.04)	35.13 (14.79)	−4.37 (−6.36–−2.39)	<0.001*	0.0044*	1.482
**FMI (kg/m^2^)**	11.85 (4.63)	10.54 (4.25)	−1.31 (−1.92–−0.70)	<0.001*	0.0044*	1.443
**VAT area (cm^2^)**	221.03 (111.12)	189.91 (114.06)	−31.12 (−54.80–−7.44)	0.015*	0.0302*	0.883
**LM (kg)**	66.42 (13.34)	65.80 (14.06)	−0.62 (−2.20–0.95)	0.397	0.3969	0.267
**LMI (kg/m^2^)**	18.86 (2.49)	18.66 (2.68)	−0.20 (−0.69–0.29)	0.382	0.3969	0.276
**ALM (kg)**	28.92 (6.33)	28.54 (6.51)	−0.38 (−1.06–0.30)	0.241	0.3208	0.376
**ALMI (kg/m^2^)**	8.62 (1.24)	8.50 (1.34)	−0.12 (−0.33–0.09)	0.222	0.3208	0.392
**BMC (kg)**	3.26 (0.71)	3.23 (0.71)	−0.03 (−0.07–0.03)	0.293	0.3520	0.334

**Abbreviations:** BW, body weight; BMI, body mass index; WC, waist circumference; TBF, total body fat; FM, fat mass; FMI, fat mass index; VAT area, visceral adipose tissue area; LM, lean mass (includes BMC); LMI, lean mass index; ALM, appendicular lean mass; ALMI, appendicular lean mass index; BMC, bone mineral content; SD, standard deviation; CI, confidence interval; BH, Benjamini-Hochberg; BH-adjusted p-values are reported for all 12 pre-post comparisons, with the false discovery rate controlled at 0.05. Descriptive statistics are presented as mean (SD). Within-group comparisons were performed using paired-samples t tests. *Statistically significant at p < 0.05.

At the aggregate cohort-level, weight reduction composition analysis indicated that 87.58% of total weight reduction was attributable to fat mass, and 12.42% was attributable to lean mass, indicating that weight reduction was predominantly attributable to fat mass. Regarding the primary outcomes, body weight decreased significantly following the intervention, with a mean change of −4.99 kg (95% CI: −7.65 to −2.34; p = 0.002, d = 1.263), corresponding to a mean percent total weight loss (%TWL) of 4.71% (Fig. 1A). Fat mass also decreased significantly by a mean of −4.37 kg (95% CI: −6.36 to −2.39; p < 0.001, d = 1.482), corresponding to a mean percentage reduction from baseline of 11.06% (Fig. 1B). In contrast, lean mass did not significantly change over the follow-up (mean change: −0.62 kg [95% CI: 2.20 – 0.95]; p = 0.397; d = 0.267), with a mean percentage change from baseline of −0.94% (Fig. 1C) (Table 2).

**Fig. 1:**
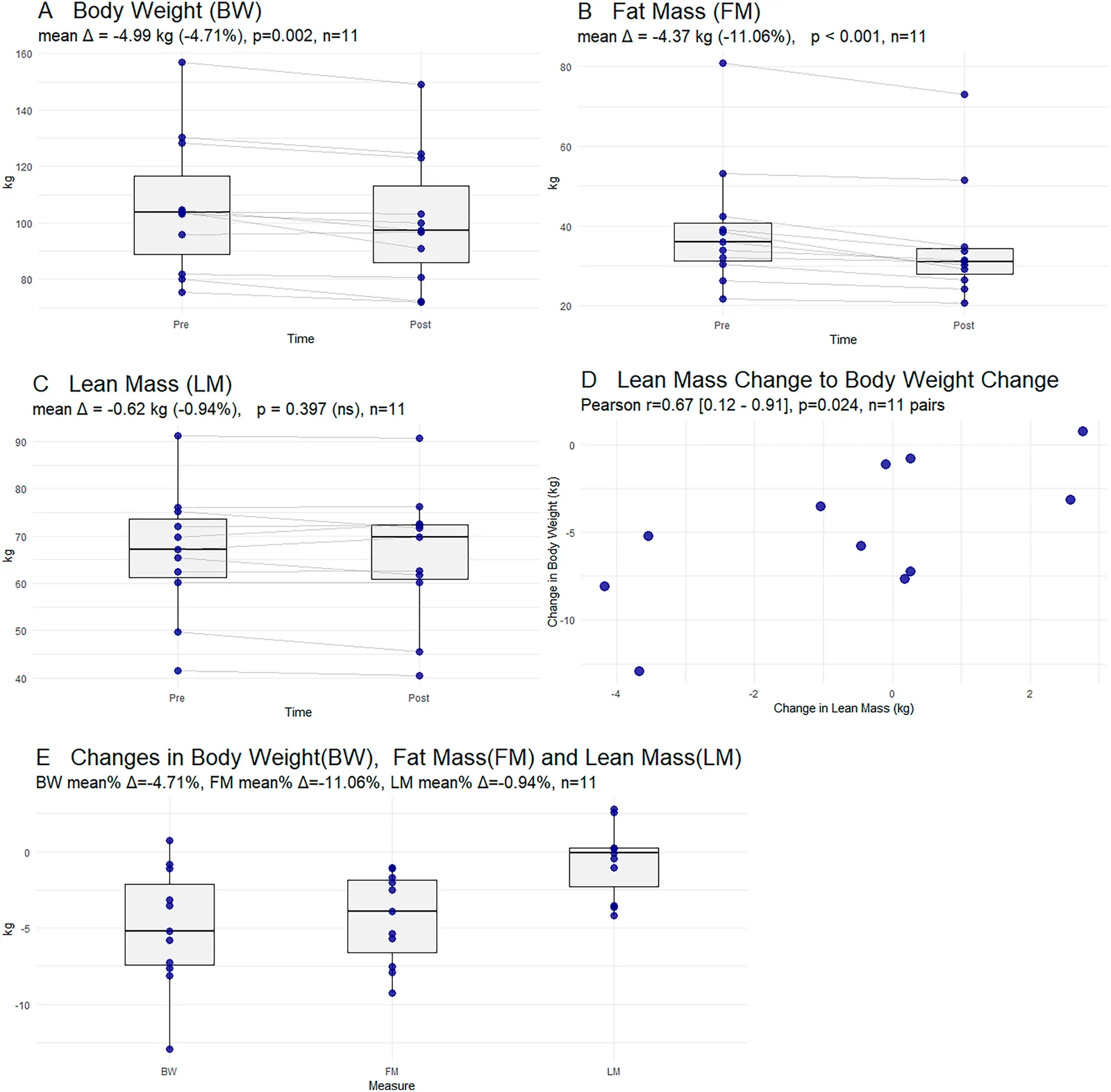
**Individual and Pre–Post Changes in Primary Body Composition Outcomes (A)** Body weight (BW) decreased significantly (p = 0.002). **(B)** Fat mass (FM) decreased significantly (p < 0.001). **(C)** Lean mass (LM) did not change significantly (p = 0.397). **(D)** Lean mass change to body weight change. **(E)** Changes in body weight (BW), Fat Mass (FM) and Lean Mass (LM). Box plots represent the median and interquartile range, and individual participant changes shown as connecting lines.

Among the exploratory secondary outcomes, BMI decreased significantly by a mean of −1.52 kg/m^2^ (95% CI: −2.37 to −0.68; p = 0.002, d = 1.207), corresponding to a mean percentage reduction from baseline of 4.79%. Total body fat percentage changed by −2.36% (95% CI: −3.56 to −1.15; p = 0.001; d = 1.310), corresponding to a mean percentage reduction from baseline of 6.44%. Fat mass index decreased by 1.31 kg/m^2^ (95% CI: −1.92 to −0.70; p < 0.001; d = 1.443), corresponding to a mean percentage reduction from baseline of 11.05%. Visceral adipose tissue area decreased by 31.12 cm^2^ (95% CI: −54.80 to −7.44; p = 0.015; d = 0.883), corresponding to a mean percentage reduction from baseline of 14.08% (Table 2).

No statistically significant changes were observed in waist circumference (mean change (95% CI) = -2.54 cm (−5.11 – 0.03); p = 0.052), lean mass index (mean change (95% CI) = -0.20 kg/m^2^ (−0.69 - 0.29); p = 0.382), appendicular lean mass (mean change (95% CI) = −0.38 kg (−1.06 – 0.30); p = 0.241), appendicular lean mass index (mean change (95% CI) = −0.12 kg/m^2^ (−0.33 – 0.09); p = 0.222), bone mineral content (mean change (95% CI) = −0.03 kg (−0.07 - 0.03); p = 0.293). The mean percentage changes from baseline for these variables were −2.20%, −1.06%, −1.31%, −1.39%, and −0.92%, respectively (Table 2). In the false-discovery-rate sensitivity analysis across all 12 pre-post comparisons, BW and FM among the primary outcomes and BMI, TBF%, FMI, and VAT among the exploratory secondary outcomes remained statistically significant after Benjamini-Hochberg adjustment (Table 2).

The positive relationship between body weight change and lean mass change was observed in Fig. 1D) (r = 0.67[95% CI = 0.12–0.91]; p = 0.024). Individual pre-post changes in primary body composition outcomes were shown in Fig. 1E.

## 4 Discussion

The primary finding of this real-world observational study was that participants experienced a 4.71% reduction in body weight and an 11.06% reduction in fat mass, whereas lean mass decreased by only 0.94% and did not change significantly during follow-up. Although previous body composition studies of semaglutide and tirzepatide have reported concurrent reductions in fat mass and lean body mass, direct comparison with these studies should be interpreted cautiously because participants in the present cohort achieved a substantially smaller mean weight reduction (4.71%) than those reported in the STEP 1 body composition analysis (15.0%) [6] and the SURMOUNT-1 DXA substudy (21.3%) [7]. Since the proportion of lean mass loss may vary with the magnitude of total weight reduction, comparisons across studies with markedly different weight reductions may be misleading. A more appropriate comparison is the placebo arm of the SURMOUNT-1 DXA substudy [7], which achieved approximately 5.3% weight reduction, closely matching the magnitude of weight reduction observed in the present cohort (4.71%). In the SURMOUNT-1 DXA substudy, approximately 75% of total weight reduction was attributable to fat mass and 25% to lean body mass in the placebo group. By comparison, 87.6% of total weight reduction in the present study was attributable to fat mass, whereas 12.4% was attributable to lean mass.

Although the observational design precludes causal inference, the lower proportion of weight loss attributable to lean mass compared with the magnitude-matched SURMOUNT-1 placebo group is consistent with a potentially more favorable body composition pattern in the context of structured exercise and individualized nutritional support. The positive correlation between changes in body weight and lean mass (r = 0.67, 95% CI 0.12–0.91; p = 0.024) further suggests that lean mass change remained related to the magnitude of overall weight reduction.

Lean tissue commonly accounts for approximately one-quarter of total weight reduction, although this proportion varies with the method and magnitude of weight reduction [25,26]. Importantly, the absence of a statistically significant reduction in lean mass in the present study should not be interpreted as evidence of complete lean mass preservation, particularly given the small sample size and variability in individual responses. Nevertheless, the relatively small proportion of total weight reduction attributable to lean mass (12.4%) may be consistent with a more favorable body composition pattern in the context of the resistance exercise and individualized nutritional support incorporated into the program. Our findings are also broadly consistent with those of the prospective real-world SEMALEAN study. After 12 months of semaglutide therapy, participants achieved a mean body weight reduction of 12.7%, accompanied by an 18.9% reduction in total fat mass. Lean mass decreased by approximately 3.0 kg during the first 7 months but subsequently stabilized, while handgrip strength improved by 4.5 kg [19]. Unlike the present study, however, SEMALEAN evaluated body composition changes during routine semaglutide therapy without a structured intervention incorporating individualized exercise prescription and dietitian-delivered protein support initiated concurrently with treatment. Although the magnitude of weight reduction was substantially greater than that observed in the present cohort, both studies demonstrated a predominance of fat mass reduction relative to lean mass loss during obesity medication therapy in real-world clinical settings.

Although Lundgren et al. examined weight reduction maintenance after an 8-week low-calorie diet, structured exercise increased lean mass and reduced fat mass, while exercise plus liraglutide preserved lean mass, with a modest +0.5 kg change, reduced fat mass by approximately 4.8 kg and body-fat percentage by 3.9 percentage points, and produced a 9.5 kg greater weight reduction versus placebo [9]. Likewise, a secondary analysis of the same trial by Jensen et al. reported that combined exercise and liraglutide treatment was associated with a 3.7 kg reduction in body weight and minimal change in appendicular fat-free mass (+0.03 kg) [10]. In the present study, appendicular lean mass did not change significantly (mean change −0.38 kg), a finding broadly consistent with these previous observations despite differences in study design, intervention, and magnitude of weight reduction. Fat mass accounted for 87.6% of total weight reduction, while the modest 4.71% total weight reduction was accompanied by a 14.08% reduction in visceral adipose tissue area.

Consistent with this observation, Neeland et al. demonstrated that liraglutide treatment resulted in a 12.49% reduction in visceral adipose tissue compared with a 1.63% reduction in the placebo group over a median follow-up of 36.2 weeks [27]. Given the strong association between visceral adiposity and cardiometabolic risk [4], the magnitude of VAT reduction observed in the present study may therefore be of clinical interest despite the relatively modest total weight reduction; however, this finding should be interpreted as exploratory. Resistance training and adequate protein intake are widely recognized as important strategies for minimizing lean tissue loss during weight reduction and are recommended in current clinical practice guidelines [15] particularly in the context of incretin-based therapy [4,5]. However, in our retrospective analysis, both supervised resistance-training attendance (1.24 sessions/week; target: ≥2) and follow-up protein intake (1.15 g/kg/day) were below the recommended targets. Despite these levels being below the recommended targets, the observed body-composition pattern remained consistent with the existing hypothesis that resistance exercise and nutritional support may help minimize lean mass loss during weight reduction. Importantly, as a real-world retrospective study, our findings reflect the exercise participation and protein intake actually achieved during incretin-based therapy rather than ideal guideline-based targets, while not establishing a causal effect.

### 4.1 Strengths

A key strength of this study is the provision of real-world evidence on DXA-derived body composition changes during obesity medication therapy combined with structured lifestyle support. The integration of individualized exercise prescription and dietitian-guided nutritional support allowed body composition changes to be characterized within a multidisciplinary treatment setting. In addition, comprehensive DXA evaluation of fat mass, lean mass, appendicular lean mass, visceral adipose tissue area, and bone mineral parameters provided detailed characterization of tissue-specific responses during weight reduction.

### 4.2 Limitations

The retrospective design, small sample size, and absence of a control group limited statistical power, precision of effect-size estimates and causal inference. A facility-specific least significant change (LSC) assessment had not been established for the DXA system; therefore, small longitudinal changes in soft-tissue measures should be interpreted cautiously. Selection and completer bias are likely because only 11 of 25 screened individuals were analyzed, and included individuals differed from excluded individuals across multiple baseline anthropometric and body-composition measures. The direction and magnitude of this bias cannot be determined. The small sample also precluded separate evaluation of semaglutide and tirzepatide and, with only two women, sex-specific analyses. Documented supervised exercise exposure varied among participants, and aerobic exercise adherence could not be consistently quantified. Standardized longitudinal muscle-strength data were also unavailable for most participants. Moreover, participants were recruited from a self-selected commercial weight management program with access to individualized exercise prescription, dietitian-delivered nutritional support, and close clinical follow-up. Consequently, these findings may not be directly generalizable to routine office-based obesity practice, where access to such multidisciplinary resources is often limited. Accordingly, the findings should be considered hypothesis-generating and require confirmation in adequately powered prospective controlled studies.

## 5 Conclusion

In conclusion, this real-world observational study demonstrated that incretin-based therapy delivered within a commercial weight management program incorporating individualized exercise and nutritional support was associated with significant reductions in body weight and fat mass, without a statistically significant change in lean mass. Although causal inference cannot be established, these findings are consistent with the hypothesis that structured lifestyle support may contribute to more favorable body composition changes during incretin-based weight reduction and warrant confirmation in adequately powered prospective controlled studies.

## 6 Clinical takeaways

• In this real-world cohort, incretin-based therapy delivered within a structured lifestyle program was associated with predominantly fat mass loss without a statistically significant reduction in lean mass.
• These findings support further investigation of structured exercise and nutritional support as potential strategies for minimizing lean mass loss during incretin-based weight reduction.
• Prospective controlled studies are needed to determine whether integrating individualized exercise and nutritional support into incretin-based obesity treatment improves body composition outcomes.

## Authors’ contributions

Meral Kucuk Yetgin conceived the study, analyzed the data, interpreted the results, and wrote the first draft of the manuscript. Nana Gletsu-Miller provided scientific advising, contributed to data interpretation, and critically revised the manuscript. Robert Chapman provided overall scientific advising. Eitan Gelber supervised the intervention procedures and contributed to data acquisition. John Snyder contributed to data acquisition and implementation of the intervention protocols. Donny Gregg and Dustin Nabhan contributed to data acquisition and delivery of the exercise interventions. All authors critically revised the manuscript, approved the final version, and agreed to be accountable for all aspects of the work.

## Ethical adherence and ethical review

The project was submitted to the Indiana University Institutional Review Board under Protocol No. 30912. Following review, the Board determined that the project did not constitute human subjects research because only retrospective, de-identified clinical data were analyzed; accordingly, Institutional Review Board approval and informed consent were not required. The clinical data were obtained from Apeiron Life (California, USA), where all participants received routine clinical care. In accordance with Apeiron Life privacy policy, only de-identified data were made available for research purposes, and the study team did not have access to identifiable participant information.

## Data availability statement

De-identified participant-level data underlying the analyses reported in this study are publicly available in Zenodo at https://doi.org/10.5281/zenodo.22287176. The deposited dataset includes the anthropometric and DXA-derived body composition data necessary to independently verify the reported analyses and weight-change composition calculations, together with a data dictionary.

## Declaration of artificial intelligence

An artificial intelligence-assisted tool was used during manuscript preparation for language refinement and correction of grammatical errors only. All scientific interpretation, data verification, statistical decisions, and final manuscript content were independently conducted, reviewed and approved by the authors. The graphical abstract was created with the assistance of OpenAI Codex (OpenAI, San Francisco, CA, USA) and was subsequently reviewed and revised by the authors for scientific accuracy.

## Funding

This study received no external funding.

## Declaration of competing interests

Eitan Gelber (Director of Assessment & Training), John Snyder (Senior Physical Performance Specialist & Sports Scientist), Donny Gregg (Senior Exercise Physiologist), and Dustin Nabhan (Chief Scientific Advisor and Consultant Sports Scientist) are affiliated with Apeiron Life, California where the study was conducted. This affiliation is declared as a potential conflict of interest.
